# Allocating intricacies: pediatric oral health spotlight in the union health and well-being budget of India

**DOI:** 10.3389/fdmed.2023.1134294

**Published:** 2023-08-01

**Authors:** Vaibhav Kumar, Rushikesh Sangle, Romi Jain, Nikhil Bhanushali, Sakshi Yadav, Ayesha Qureshi, Harshal Tandel, Pranjal Mhatre

**Affiliations:** ^1^ Department of Public Health Dentistry, GD Pol Foundation YMT Dental College, Navi Mumbai, India; ^2^ Department of Public Health Dentistry, TPCT’s Terna Dental College and Hospital, Navi Mumbai, India; ^3^ Department of Research, Suitradhaar Strategies Pvt Ltd, Kolkatta, India

**Keywords:** union health budget, Indian healthcare, oral health, national oral health comprehensive intervention program for children, national oral health program

## Introduction

### Healthcare in the union health and well-being budget

Health is a state of physical, mental, and social well-being and not just the absence of disease and infirmity. Healthcare services help reduce mortality rates, keep diseases in check, and raise life expectancy, which play a substantial role in the economic growth of a country (1). The Union Budget 2021 was prescribed for the first time with consideration to holistic health care and well-being, yet, as per National Health Profile (NHP) data of 2019, India spends just over 1% of its GDP on public health, which is drastically low considering the country's population, demographics, and ever-increasing disease burden (2). However, with increased awareness about healthcare in the post-pandemic era, this trend appears to be shifting. For the financial year 2022–23, Rs 2,23,846 crore was allocated towards healthcare in the budget presented, which was 137% higher than the preceding year (Rs. 94,452 crore) (3). The Union Budget 2023–24 has been called the first of “amrit kaal”, or the elixir era, aims to achieve the goal of India becoming a developed country in the next 25 years. The Ayurveda, Yoga and Naturopathy, Unani, Siddha and Homeopathy (AYUSH), Health and Family Welfare, and Finance ministries of India are responsible for allocating the entire Union Health Budget. For 2023–2024, the total budget for health across all three ministries is Rs. 1,06,654 crores. Of the total Union Health Budget, about 2.3% is allocated to pediatric healthcare (3).

## Upgraded contrivances

### The Prime Minister Atmanirbhar Swasth Bharat Yojana scheme

This scheme, with an outlay of 64,180 crores over 6 years, will consist of 15 health care emergency centers and two mobile hospitals, establishing critical care hospital blocks in 602 districts and 12 central institutions, strengthening national centers for disease control and its five branches and 20 metropolitan health surveillance units, providing support for health and wellness centers with integrated public health care labs in all districts and 3,382 block public health units in 11 states, and the setting-up of nine bio-safety level three laboratories and four regional National Institutions for Virology (4).

### Swachch bharat, swasth bharat

This scheme will be implemented with an allocation of 1,41,678 crores over a period of 5 years from 2021 to 2026, merging the Supplementary Nutrition Programme and the Poshan Abhiyan and is aimed at the cleaning of fecal sludge, wastewater treatment, source segregation of garbage, a reduction in the use of plastic, a reduction of air pollution, and bioremediation of all legacy dump sites while also improving the nutritional outcomes in 112 districts (5).

### The Jal Jeevan Mission

This scheme will be implemented over 5 years with an outlay of 2,87,000 crores stressing the importance of clean water, sanitation, and a clean environment whilst providing the water supply in all 4,378 urban local bodies, 2.86 crores of household tap connections, and liquid waste management in 500 Atal Mission for Rejuvenation and Urban Transformation cities (6).

### The Pradhan Mantri Swasthya Suraksha Yojana (PMSSY)

The budget allocated to establishing a new All India Institute of Medical Sciences (AIIMS) and refining the existing Government Medical Colleges has been reduced by Rs. 517 crores from last year (7).

### The Pradhan Mantri Jan Arogya Yojana (PMJAY)

This scheme's budget allocation has doubled from Rs. 3,100 crores in 2020–21 to Rs. 6,400 crores in 2021–2022 (8). Through this scheme, the Government of India aims to establish a public health insurance fund for the economically weaker sections of society. It takes into account the inability of the population to access basic healthcare. However, no mention of oral health insurance has been made. This scheme especially lacks attention to pediatric health and, more so, to pediatric oral health.

### The national AIDS and STD control programme

Unfortunately, the budget for this initiative remains unchanged at Rs. 2,900 crores (9). India, with 2–3 million individuals infected with HIV, requires significant attention and funds to manage highly prevalent STIs. In children, the Maternal-to-Child Transmission rate of HIV is about 40%–45%. The number of children enrolled in the HIV National Program are expected to be above one lakh but approximately only 30% of them are supposedly on Anti-Retroviral Therapy (10). Hence, this program is inadequate in achieving the required amount of resources and ensuring their proper distribution.

**Figure 1:**
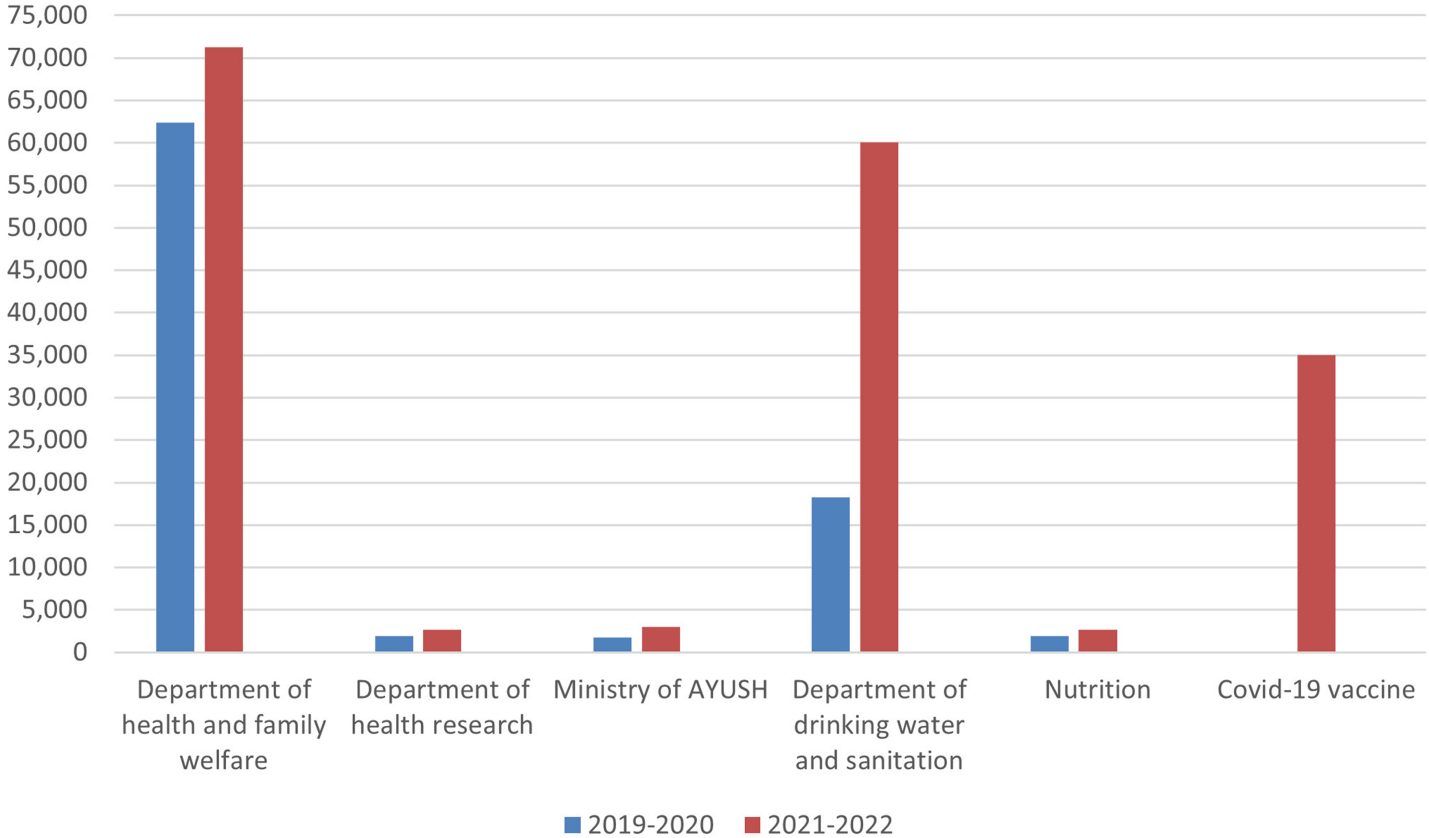
A comparative chart of the healthcare budget distribution between the years 2019–20 and 2021–22.

Figure 1 depicts the comparative analysis of the health care budget distribution between the years 2019–20 and 2021–22 across various health departments in India. Compared to the financial year 2019–20, the allocation of funds to the Department of Health and Family Welfare showed a significant rise. This trend was further observed in the allocation of the budget to the Department of Drinking Water and Sanitation. In the financial year 2021–22, a large sum of the Union Budget was allocated for the development and distribution of the COVID-19 vaccine.

## India placed against its global contemporaries: the Ying and Yang

Total expenditure as a percentage of GDP in India is as low as 1.26%. It has been reported that India has one of the lowest public healthcare budgets in the world; countries like the United Kingdom, the Netherlands, New Zealand, Finland, and Australia spend over 9% of their total GDP on public healthcare, Japan, Canada, France, Germany, and Switzerland spend about 10%, and the United States 16%. In India, only 25% of the population has access to sanitation, and the use of diagnostic testing is almost in India. Though there has been a considerable increase in the Union Budget allocated to the healthcare sector from Rs 62,659.12 in 2019–20 to Rs 69,000 crore in 2020–21, there still seems to be scope for betterment (11). Out-of-pocket payments account for 70% of healthcare costs in India, whereas in the US these account for around 10%–12%.

## Prioritising non-communicable diseases: need of the hour

In India, 63% of deaths are due to non-communicable diseases (NCDs) and 11% due to injuries. And yet the government spends less than 0.5% of its GDP on NCDs and so the states with high poverty levels have a low per capita expenditure on NCDs (12). When applied to the dental sector, this is especially true in cases of dental caries. According to a systematic review conducted by Shah et al., 49.6% of the children (approximately 100 million children) below the age of five years in India live with untreated dental caries. This substantially increases the burden of disease in the population and highlights the need for effective implementation of preventive strategies with early interventions such as fluoride application, pit and fissure sealants, etc., in the National Oral Health Policy (13).

## The inverse care law: the Pandora's box

While the Indian healthcare sector is divided into public and private, the private healthcare segment in India is mainly focused on urban centers, leading to the unequal distribution of services, with 75% of the healthcare infrastructure concentrated in urban areas where only 27% of the total Indian population resides. Only 11% of sub-centers, 13% of Primary Health Centers (PHCs), and 16% of Community Health Centers (CHCs) in rural India meet the Indian Public Health Standards (IPHS). Only one allopathic doctor is available for every 10,000 people and one state-run hospital is available for every 90,000 people. As per the 2017–18 budget announcement, 1,50,000 Health Sub Centers and Primary Health Centers are to be transformed into Health and Wellness Centers (AB-HWCs) by December 2022 to provide Comprehensive Primary Health Care (CPHC) to ensure healthcare for all (14). The PHCs in India are the primary point of contact for services regarding non-communicable diseases. Therefore, oral health promotion, check-ups, and appropriate referral as well as screening for chronic non-healing ulcers is an essential function of the PHCs (15).

## Oral health: the “international neglect”

The Union Health and Well-Being Budget 2021 was announced for the first time as a holistic presentation of healthcare needs, assimilating and amalgamating traditional and modern healthcare delivery systems and needs. Whilst allocating significant funds toward the AYUSH (Ayurveda, Yoga, Unnani, Siddha and Homeopathy) framework, a disappointing zero percent of the GDP has been allocated towards oral and dental care needs. No distinct consideration was given to oral health care under the Union Budget 2021–22 despite the schema of the Common Risk Factor Approach proving a strong relationship between the departure from oral health and its myriad links with systemic illness.

**Figure 2:**
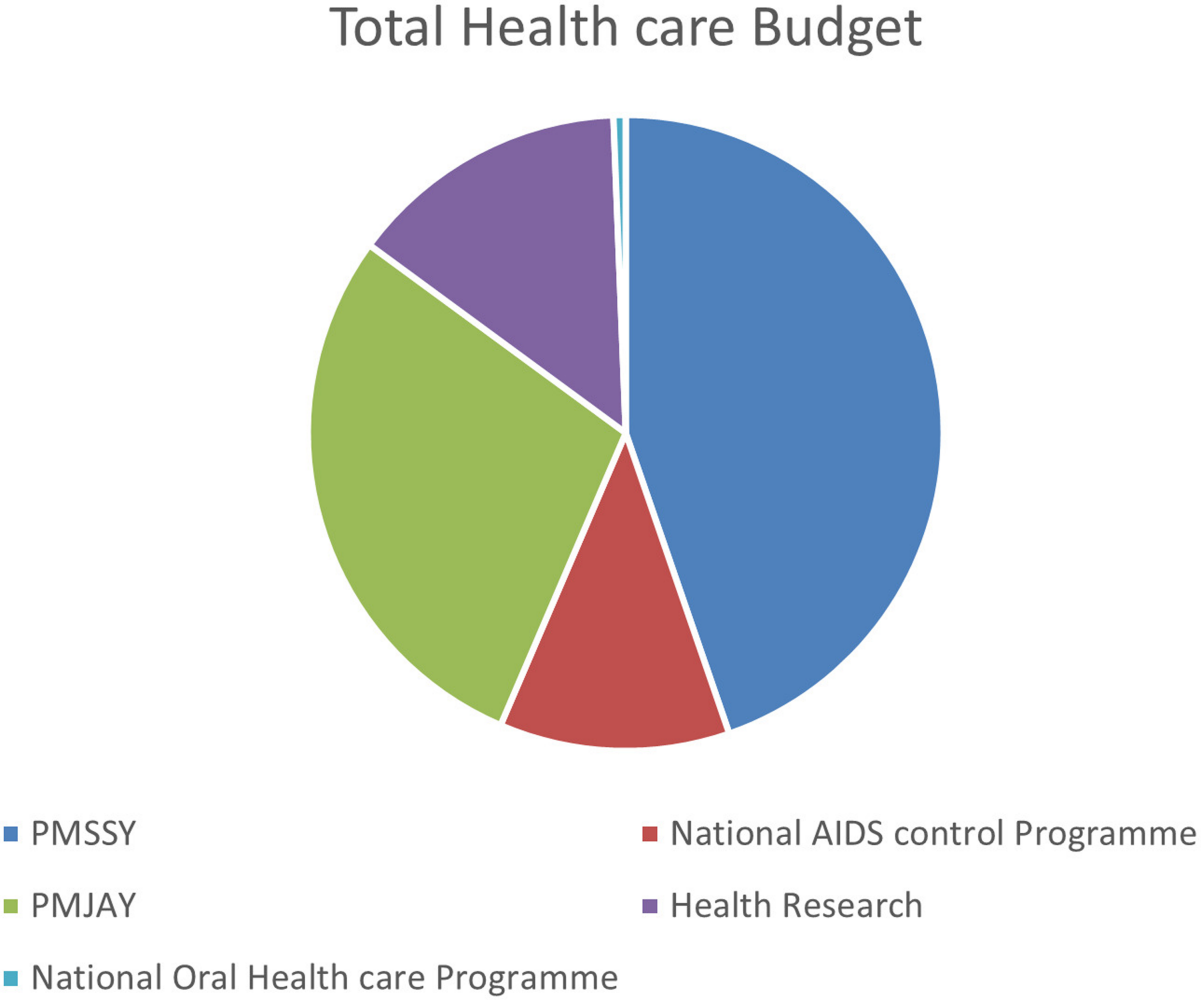
A pictorial representation of the major allocations in the healthcare budget.

Oral health is an integral component of general health yet oral diseases still remain a burden for developing countries like India, especially among the rural population (16). Amongst emerging countries, China enjoys a relatively favorable dental health status and, amongst high-income countries, South Korea exhibits the best dental health status (17). In India, poor oral health status maybe primarily attributed to ignorance among the masses. According to one study (Mathur 2021), 95% of adults in India suffer from gum disease, 50% of citizens do not use a toothbrush or toothpaste, and 70% of children under the age of 15 have dental caries (18), which proves that the burden of oral diseases is on the rise, with oral health being an issue of “international neglect” by policymakers (19). The capacity of the existing health system to overcome these challenges is uncertain; as shown in Figure 2, the budget allocation to oral health care seems negligible compared to other policies. There is no specific allotment for the oral healthcare of the pediatric population as the majority of oral diseases can be controlled if intercepted at this growing age. To improve the system and bring about a policy change, a systematic analysis of the existing oral health system is necessary.

## National oral healthcare programme: the oral health delivery fabric of India – thrusting beyond boundaries

The NOHP is a stint of hope at acknowledging the gravity of oral health care. Praiseworthy initiatives like establishing 85 Muskaan clinics providing free dentures to anyone above the age of 65 years is a part of the Danta Bhagya Yojane, the national oral health policy which was drafted in February 2021 as a part of this initiative. The policy has appreciated the importance of equity, integration, community participation, gender, prevention and promotion, and research which would help in addressing the oral disease burden in India. The Rashtriya Bal Swasthya Karyakram which is a milestone so set that appraising the overall quality of life of children which involves screening of children from birth to 18 years for defects at birth, diseases, deficiencies, and developmental delays. The National Cancer and Tobacco Control Program, National Rural Health Missions, and School Health Program are other budding prospects for efficient delivery of oral health to the population.

Centers for oral health care like the PGI Chandigarh launching the E-RCTC- a joint initiative of PGIMER Chandigarh and the Union to strengthen the National Control Tobacco Programme (NTCP) has been launched. The Maulana Azad Institute of Dental Science (MAIDS) which has fabricated the Mobile Dental Clinic Project and Antitobacco cell, Centre for Dental Education and Research (CDER) is a part of Cochrane Oral Health's Global Alliance and is the National Centre of Excellence for Implementation of the National Oral Health Programme and the World Health Organization (WHO) Collaborating Centre for Oral Health Promotion have also been devised. These schemes have the primary objective of narrowing the rural-urban gap in oral healthcare with a definite budget allocation for the same, thereby increasing the utilization of public oral health facilities and community-based awareness by at least 50% per district by 2030, establishing baseline data for the oral disease burden of the country by 2025, and reducing morbidity and mortality from them by 15% by 2030. To ensure a district-level electronic database of information on health system components by 2025 while integrating oral health information architecture and exchanges between district and primary health centers by 2030. The aims of these initiatives is to eventually strengthen the oral health care system. As no specific care or onus is given to children between 6 or 12 years of age, it becomes essential to gauge how to cease the sustained degradation of oral health care in places where access and resources are inadequate through a common risk factor approach.

### Importance of pediatric oral health and incremental care

While many nations have seen improvements in a variety of oral health metrics, India has not. According to a biannual multi-centric oral health study undertaken by the Ministry of Health and WHO in India in 2007–2008, dental caries prevalence among 12-year-olds ranged from 23% to 71.5%. A systematic review that was released in 2018 indicated that 49.6% of Indian children under the age of six had untreated dental caries (21). The number of children with untreated dental caries is roughly 10 crores if this percentage is extrapolated to children under the age of six. These figures imply that an incremental care method is more appropriate for a developing nation like India because it is periodic care which provides the children with priority dental treatment in a step-by-step manner. The procedure has its own benefits, although it is very occasionally used in complete projects. However, by identifying the needy, the treatment providers, the funding source, and using modern data processing techniques to collect and study the information, one can better understand the many factors involved, possibly make better predictions, and make better decisions about the allocation of resources to solve the problems in health care and achieve the maximum benefit of using the straightforward procedure of providing dental care incrementally to cover the children who will be the country's future citizens.

### The role of the union budget of India and its influence on international agencies

The yearly upward trend in the allotment of funds to the healthcare sector is a crucial indicator of the progressive development in the country. A developing nation such as India serves as a template for international agencies as well as other developing nations to better understand the direct effects of increased financial aid on the incidence and prevalence of diseases in the population. Through the betterment in overall mortality and reduced burden of disease in the population, India serves as a great example to other countries and healthcare agencies to formulate an effective plan of action towards achieving global health.

## Conclusion

The essence of this communication lies in the fact that there has been considerable progress in the Government’s budget allocators and policymakers' consideration towards oral health and health, in general. Although an integral part of national upliftment, the oral health system of India is deficient in many aspects. The reorientation of oral health services is required to counteract the problems faced due to various oral diseases. Encouraging the system to bring about a change and help in providing attention to a systematic analysis of the oral health care system is necessary for the eradication of existing oral diseases.

About 50% of children in India under the age of six years live with untreated dental caries. This study throws light upon the need for the allocation of funds and the imposing of policies for the betterment of pediatric oral health and general oral health, and highlights the existence of numerous national health schemes devoted towards pediatric oral health such as Danta Bharat Yojna and Rasthriya Bal Swasthya Karyakram. The Union Health Budget allocates an average of 2.3% towards the pediatric health sector. However, the intricacies of this budget towards pediatric oral health is unknown. It is indeed high time to give due diligence to the importance of oral healthcare in India. The criticality and exigency of the deteriorating oral health status of children should not be undermined, as preventive care and pediatric oral care should be given as equal status as curative and restorative care.

The authors declare that this review is an independent opinion and that the authors do not support any particular government or political party or organization.
